# Analysis of risk factors for cesarean scar diverticulum: A STROBE-compliant case-control study

**DOI:** 10.1097/MD.0000000000025757

**Published:** 2021-04-30

**Authors:** Songjun Liu, Linlin Chen, Guang Zhu, Yupei Shao, Shuqian Yu, Wu Li, Wen Lv

**Affiliations:** Department of Gynecology, Tongde Hospital of Zhejiang Province, Hangzhou, Zhejiang, PR China.

**Keywords:** cesarean section, diverticulum, risk factors

## Abstract

The purpose of this study was to explore the relevant factors that affect the risk of cesarean scar diverticulum (CSD).

A retrospective, case-control study was designed among women with a history of cesarean section (CS) who were admitted in Zhejiang Tongde Hospital from January 2017 to December 2019. Women with missing information were excluded. The basic clinical characteristics and the risk factors for CSD were assessed using univariate analysis and multivariate logistic regression analysis.

A total of 216 women were analyzed, including 87 patients with CSD and 129 cases without CSD as control. Significant differences in number of CS, trial of labor (elective or urgent CS), CS interval, uterine position, intraoperative hemorrhage, and dysmenorrhea between CSD group and control group (*P* < .05). Multivariate logistic regression analysis showed that number of CS, trial of labor, interval of CS, and uterine position were independent risk factors of CSD.

In women with a history of CS, multiple cesarean deliveries, elective CS, cesarean interval of less than 5 years, and retroflexed position of the uterus may be associated with an elevated risk of CSD.

## 1 Introduction

Cesarean scar diverticulum (CSD), also known as “cesarean scar defect,” “isthmocele,” “niche,” “pouch,” or “cesarean scar dehiscence,” and is characteristic of anterior uterine isthmus defect at the site of previous cesarean section (CS).^[1]^ The incidence of CSD has been reported to vary from 24% to 84% by using transvaginal ultrasound or sonohysterography.^[2]^ Women with CSD may suffer from prolonged menstrual period, menorrhagia, dysmenorrhea, or secondary infertility.^[3,4]^ Additionally, CSD may cause life-threatening complications during subsequent pregnancy, including cesarean scar pregnancy, placenta previa, placenta accreta, and even uterine rupture.^[5]^ In consideration of CSD-related complications, it is important to identify possible risk factors contributing to the formation of CSD. In this study, we designed a retrospective, case-control study to explore the risk factors of CSD for the sake of providing some useful information in clinic and finding ways to reduce the development of CSD.

## 2 Methods

### 2.1 Patients selection and study design

Retrospective analysis of case records was carried out in Tongde Hospital of Zhejiang Province between January 2017 and December 2019, including patients diagnosed of CSD and patients with history of CS but not sonographically presenting with CSD.

**Figure 1:**
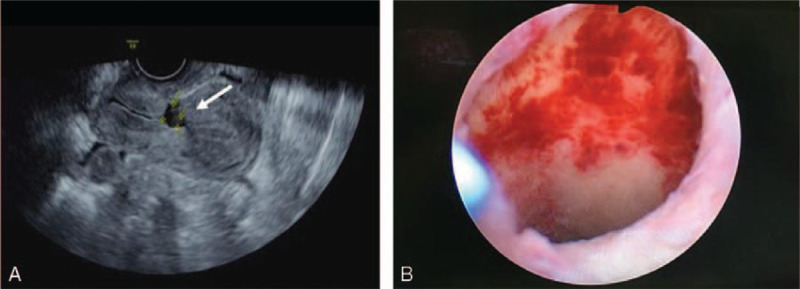
Diagnosis of CSD. A. Transvaginal ultrasound image of CSD: a wedge-shaped distortion in the integrity of the uterine incision scar was confirmed as CSD (white arrow). B. Hysteroscopy showed the “isthmocele” in the superior third of the cervical canal.

Patients were enrolled into CSD group for the following eligibility criteria:

1. a history of at least one CS performance,
2. firstly, diagnosed of CSD by transvaginal ultrasound and further confirmed by hysteroscopy (Fig. 1),
3. no history of uterine surgeries other than CS.

The key exclusive criteria included:

1. a history of previous menstrual irregularity before CS,
2. use of intrauterine device,
3. evidence of other uterine diseases, such as leiomyoma, endometrial polyps, hyperplasia, or adenomyosis,
4. coagulatory disorders.

**Figure 2:**
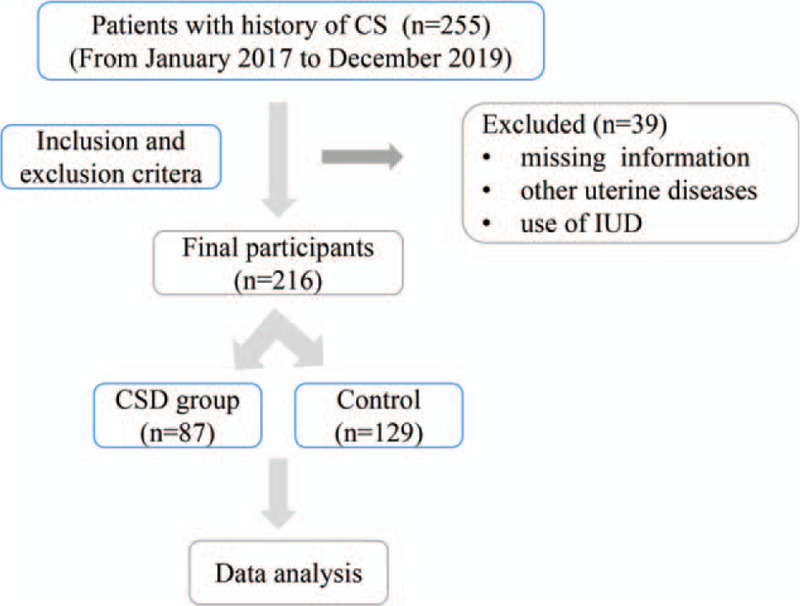
Flow chart of the study.

Patients in control group were designed to be matched in an approximately 2:1 ratio to index CSD cases with respect to patient age and gestational weeks. However, during January 2017 and December 2019, not all the patients met the eligibility and exclusive criteria. Meanwhile, subjects with missing information were also excluded. Subsequently, a total of 87 cases and 129 controls were finally included (Fig. 2).

### 2.2 Data collection

Data for this study were collected prospectively in the Institutional Review Board approved database of Tongde Hospital of Zhejiang Province. Subsequently, patients in CSD group who underwent laparoscopic were identified retrospectively. Using electronic medical records and telephone follow-up, the following data from the clinic and inpatient services were collected for analysis: age, gestational week, number of CS, CS interval, dysmenorrhea, and hemorrhea.

### 2.3 Statistical analysis

Statistical analysis was performed by using SPSS 23.0. Continuous data were presented as mean ± standard deviation and percentages. Two-sample *t* test was used to compare clinical data of age and gestational week. Categorical variables were analyzed using Chi-Squared test or Fisher exact test. And multivariate log-binomial regression analysis was performed to identify odds ratio with 95% confidence intervals. *P* < .05 (2-tailed) was considered statistically significant.

## 3 Results

### 3.1 Characteristics of patients

As Table 1 presented, the study admitted a total of 87 CSD cases with a mean age of 23.4 ± 13.6 years. The control group was consisted of 129 women with a mean age of 22.6 ± 14.8 years. Majority of CSD cases (56 participants, 64.4%) underwent 2 times of CS, and 9 (10.3%) participants received 3 times of CS. While in the control group, none has undergone triple CS and most women (72.1%) only had experienced one CS. There were 40.2% patients with CSD and 24.1% in the control group suffering from dysmenorrhea, while intraoperative hemorrhage was relatively a low incidence with 10.3% in CSD group and 2.4% in the control group.

**Table 1:** Baseline characteristics.

	CSD group	Control group	
Variable	n = 87	n = 129	*P* value
Age (yr) (Mean ± SD)	23.4 ± 13.6	22.6 ± 14.8	.230^∗^
Gestational age (wk)			
(Mean ± SD)	38.1 ± 3.8	38.4 ± 2.6	.340^∗^
Uterine position (n, %)			
Retroflexed	75 (86.2%)	31 (24.1%)	.000^†^
Anteflexed	12 (13.8%)	98 (75.9%)	
Number of CSs (n, %)			
1	22 (25.3%)	93 (72.1%)	.000^‡^
2	56 (64.4%)	36 (27.9%)	
3	9 (10.3%)	0 (0%)	
Interval of CS (n, %)			
≤5 yr	50 (76.9%)	20 (55.6%)	.042^†^
>5 yr	15 (23.1%)	16 (44.4%)	
Trial of labor (n, %)			
Yes	28 (32.2%)	88 (68.2%)	.000^†^
No	59 (67.8%)	41 (31.8%)	
Intraoperative hemorrhage (n, %)			
Yes	9 (10.3%)	3 (2.4%)	.015^†^
No	78 (89.7)	126 (97.6%)	
Dysmenorrhea (n, %)			
Yes	35 (40.2%)	31 (24.1%)	.016^†^
No	52 (59.8%)	98 (75.9%)	

### 3.2 Risk factors associated with CSD development

Significant differences were found between CSD group and the control group, including uterine position, number of CSs, cesarean interval, trial of labor, intraoperative hemorrhage, and clinical symptom of dysmennorrhea (all *P* < .05, Table 1). No obvious difference was seen in age and gestational week (*P* > .05).

The crude and adjusted effects for risk factors of CSD have been displayed in Table 2. In the adjusted analyses, cases with multiple CS were seen to 3 times increased risk of attaining CSD compared to women with single CS. Besides, results also showed that women whose uterus was retroflexed and whose interval of CS was within 5 years had higher risk of developing CSD. We did not find a significant association between intraoperative hemorrhage and CSD. The adjusted analysis for association between trial of labor and CSD indicated that women undergoing elective CS tended to have 4.5 times increased risk of CSD formation than those who had a trial of labor but ended up to CS.

**Table 2:** Multivariate logistic analysis for risk factors for development of CSD.

Variable	β	SE	Wald *χ*^*2*^	*P* value	Adjusted OR (95%CI)	Crude OR (95%CI)
CS time (1 CS=0, 2, or 3 CS = 1)	1.121	0.551	4.131	.042	3.067 (1.041–9.035)	7.188 (3.896–13.262)
Dysmenorrhea (No = 0, Yes = 1)	0.419	0.455	0.849	.357	1.520 (0.624–3.707)	2.128 (1.181–3.834)
Hemorrhage (No = 0, Yes = 1)	1.436	0.879	2.672	.102	4.204 (0.751–23.524)	4.846 (1.273–18.449)
uterine position (Anteflexed = 0, Retroflexed = 1)	3.132	0.465	45.461	.000	22.924 (9.223–56.978)	19.758 (9.511–41.043)
Interval of CS (>5years or without second CS = 0, ≦5years = 1)	1.158	0.575	4.064	.044	3.184 (1.033–9.819)	7.365 (3.888–13.950)
Trial of labor (Yes = 0, No = 1)	1.506	0.426	12.495	.000	4.511 (1.956–10.399)	4.214 (2.360–7.526)

## 4 Discussion

CS rate has increased apparently in recent decades with almost one-third of women undergoing CS delivery worldwide.^[6]^ And 2-child policy in China also accelerate the incidence of CS. According to an observational study, the rate of CS increased from 29% in 2008 to 46.7% in 2016 in China.^[7]^ Owing to the increasing CS rate, the prevalence of CSD is also increasing. Women with CSD may suffer from abnormal uterine bleeding, dysmenorrhea, and serious obstetric complications. Previous studies noted that risk factors leading to poor CS scar healing may be divided into 4 categories, including closure technique, development of lower uterine segment or location of the incision, wound healing, and miscellaneous determinants.^[2]^ However, the exact etiology of CSD still remains to be clarified.

Despite that there is still lack of uniform diagnostic standard for CSD, transvaginal ultrasound is usually used as the first line appliance for the noninvasive examination of CSD.^[8]^ Other examination techniques include magnetic resonance imaging, hysteroscopy, and hysterosalpingography. Surgical treatment options for women with CSD include transvaginal diverticulum repair, hysteroscopy, and laparoscopy repair.^[9]^ In this study, women who initially diagnosed with CSD by ultrasound were further confirmed by hysteroscopy and underwent laparoscopic repair combined with hysteroscopy, an approach effective for anatomic correction, symptom relief, and fertility restoration. However, surgical intervention may cause secondary trauma and operative complications, such as intraoperative bleeding, infection, or adhesion. Thus, it is of vital importance to find effective ways to minimize the formation of diverticulum. Understanding the risk factors of CSD development may be helpful.

In the present study, we found women with retroflexed uterus were being at risk of CSD development. These findings are comparable with those of a study conducted by Tang et al where the risk of CSD development was increased in retroflexed uterus with OR of 6.315.^[10]^ Previous studies have suggested that the easier formation of CSD may be explained by the phenomena of reduced blood perfusion and oxygenation in a retroflexed uterus due to higher degree of mechanical tension of the lower uterine segment.^[11,12]^

Apart from uterine position, our study also presented an increased risk of CSD among patients undergoing multiple CS. Similarly, studies done by Ofii-Yebovi and Wang also reported the association between the number of CS and CSD.^[13,14]^ Repeated CS can cause repeated trauma to the isthmic wall and influence the wound healing process. Contrary to Chen study, we found that short interval of CS was another variable related to deficient CS scar. Chen et al, found CSD group has more cases (14/24, 58.33%) with CS interval ≧5 years.^[12]^ Previous study indicated that the histologic healing of the CS scar took a minimum of 6 months,^[15]^ so we assumed that the scar in women with a short interval of CS may not be healed thoroughly. Anyway, further studies are needed to verify the relationship between CSD and the interval of CS.

Besides, the relationship between timing of CS and CSD is also inconsistent. Our study noted that elective CS was a risk factor for the development of CSD which was in line with Chen study.^[12]^ The possible cause was assumed to be that the lower uterine segment in women with elective CS formed not as good as that in laboring and it is difficult to drain the uterine cavity and coalesce the incision if cervix was not dilated before CS was finished. However, some studies showed no significant difference in elective and emergency cesarean delivery.^[16]^ Oser et al found cervical dilation and station of the presenting fetal part may raise the risk of larger niches.^[10]^ While Yazicioglu et al reported that less cervical dilation was a risk factor for niche formation.^[17]^ The contributing factors associated with these differences may include study samples and cervical dilation degree. More large scale studies are needed to verify the effects of above determinants.

Nevertheless, there are limitations. Due to lack of some medical records, some potential risk factors have not been taken into account, including suturing technique, peripartum infection, and other clinical symptoms. Second, lack of randomization and the limited numbers of patients in each group may mask bias.

## 5 Conclusion

This study indicated that multiple cesarean deliveries, elective CS, cesarean interval of less than 5 years, and retroflexed position of the uterus may be associated with an elevated risk of CSD. Eligible strategies can be taken by clinicians to minimize the formation of CSD, including avoiding multiple CSs, allowing trial of labor, avoiding elective CSs, and educating women not being in rush to get pregnant after CS. Nevertheless, considering the relatively small samples of our study, more data are still needed to testify our conclusion.

## Author contributions

**Conceptualization:** Songjun Liu, Wen Lv.

**Data curation:** Linlin Chen, Guang Zhu.

**Formal analysis:** Songjun Liu.

**Methodology:** Guang Zhu, Yupei Shao.

**Software:** Linlin Chen.

**Validation:** Shuqian Yu.

**Writing – original draft:** Shuqian Yu, Wu Li.

**Writing – review & editing:** Songjun Liu, Wen Lv.
